# Intramedullary core decompression combined with endoscopic intracapsular decompression and debridement for pre-collapse non-traumatic osteonecrosis of the femoral head

**DOI:** 10.1186/s13018-022-03477-8

**Published:** 2023-01-02

**Authors:** Yan Zhao, Guangyang Zhang, Qichun Song, Lihong Fan, Zhibin Shi

**Affiliations:** Department of Orthopaedics, The Second Affiliated Hospital of Xi’an Jiaotong University, 157 Xiwu Road, Xi’an, 710004 Shaanxi Province People’s Republic of China

**Keywords:** Non-traumatic osteonecrosis of femoral head, Intramedullary core decompression, Intracapsular decompression, Arthroscopic debridement, Hip arthroscopy

## Abstract

**Background:**

The effect of core decompression on the treatment of pre-collapse non-traumatic osteonecrosis of the femoral head (ONFH) is still limited. This study aimed to explore the efficacy of core decompression combined with intra-articular decompression (debridement of the hip joint and incision of the hip capsule) under hip arthroscopic guidance in patients with pre-collapse ONFH.

**Methods:**

The clinical data of 101 patients with pre-collapse ONFH were analysed retrospectively. Sixty patients (80 hips) received small-diameter multi-channel core decompression alone in first half review period (group B). Forty-one patients (59 hips) were treated with small-diameter multi-channel core decompression combined with intra-articular decompression under hip arthroscopy guidance in second half review period (group A). The surgical duration; intraoperative bleeding; intra-articular pressure(IAP) before and after surgery; length of hospital stay; hospitalisation expenses; visual analogue scale (VAS) score before, 1 week, 4 weeks, 3 months and 1 year after surgery; and Harris score of the hip joint before, 3 months and 1 year after surgery were recorded and compared between group A and group B. X-ray examination was performed every month to observe the collapse of the femoral head within 2 years after surgery, which was compared using the Kaplan–Meier survival curve analysis.

**Results:**

When the two groups were compared, the surgical duration was longer and hospitalisation expenses were higher in group A than in group B (*P* < 0.05). However, the VAS score and the Harris score of the hip joint after surgery improved significantly compared with those before surgery (*P* < 0.05), which were more apparent in group A than in group B (*P* < 0.05). X-ray examination revealed that 6 hips in group A and 22 in group B received femoral head collapse at the 2-year follow-up. The survival rate of the femoral head in group A was significantly higher than that in group B (*P* < 0.05).

**Conclusion:**

Small-diameter multi-channel core decompression combined with intra-articular decompression (debridement of the hip joint and incision of the hip capsule) under hip arthroscopic guidance for treating early ONFH can more effectively alleviate joint pain, improve joint function and delay ONFH progression.

## Introduction

Non-traumatic osteonecrosis of the femoral head (ONFH) is a joint dysfunction induced by the interruption or impairment of the blood supply to the femoral head due to corticosteroid therapy, alcohol abuse and idiopathy, resulting in the death of bone cells and bone marrow components and subsequent repair, which is common in young and middle-aged individuals [1]. Untreated early ONFH can result in structural change and femoral head collapse, which may lead to degenerative and destructive changes in the hip joint even suffering total hip arthroplasty (THA) [2]. However, THA still has time limit and complication due to wear and tear, especially in young patients with functional needs [3]. Therefore, we hope that the collapse of the femoral head can be prevented by enhancing bone repair, and even the occurrence of THA can be avoided or delayed.

Although the pathophysiology of ONFH has not been fully elucidated, most researchers believe that early ischaemia is the main mechanism [4]. Therefore, the key to treating ONFH is to restore the blood supply to the femoral head and promote the formation and repair of normal bone tissues [5]. For early ONFH, core decompression is the most common surgical procedure for early intervention in osteonecrosis [6, 7]. However, when these large-diameter augers are used for multiple drilling to weaken or penetrate the femoral head, the articular cartilage and articular processes are damaged, accompanied by various complications, such as articular cartilage invasion and subtrochanteric fractures [8]. Recently, the use of multiple small drill holes and 3-mm Kirschner wires could achieve core decompression, which decreases the incidence of complications while reducing the intramedullary pressure [9, 10].

Previous studies have shown that intramedullary pressure and intra-articular pressure (IAP) are high when femoral head necrosis occurs, and elevated IAP can also lead to decreased arterial blood flow and blocked venous reflux [11, 12]. The pressure in the medial and lateral femoral circumflex arteries ranges from 40 to 80 mmHg; the retinacular arteries that pass through the joint capsule wall are the branches of the medial and lateral femoral circumflex arteries with low pressure [13]. The normal intramedullary venous pressure of the femoral head ranges from 10 to 20 mmHg [14]. The increase in IAP can lead to insufficient arterial perfusion and blocked venous reflux of the femoral head, which may cause an ischaemic–hypoxic state of bone cells, resulting in osteonecrosis [15]. Some studies have demonstrated that joint capsule incisions can significantly decrease the pressure in the joint cavity, thereby improving the blood flow and reducing the incidence of ONFH [16, 17]. Hip arthroscopy can directly deal with the factors that increase IAP [18]. Further, it can cut open the joint capsule and help reduce IAP. Thus, arthroscopic adjuvant therapy is a promising surgical method that can provide safe, accurate and minimally invasive decompression [19].

Briefly, the increased intramedullary pressure and IAP of the femoral head may lead to femoral head ischaemia and aggravate ONFH. Therefore, this study attempts to reduce the femoral head pressure, increase venous reflux, improve the blood supply to the femoral head, reverse the ischaemic state of the femoral head and enhance the success rate of hip preservation via treatment with intra-articular decompression combined with intramedullary decompression of the femoral head.

## Methods

### Subjects and grouping

Overall, 198 patients with early ONFH who were treated in our hospital (The Second Affiliated Hospital of Xi’an Jiaotong University) from January 2015 to December 2019 were analysed retrospectively, and 101 patients who met the inclusion criteria were included. The inclusion criteria were as follows: patients (1) who meet the diagnostic criteria of early ONFH with surgical indications including pain and/or limited joint function [20, 21], (2) with Association Research Circulation Osseous (ARCO) stages I and II [6], (3) aged 18–65 years, (4) who signed informed consent for surgery and (5) with a complete follow-up of > 2 years. The exclusion criteria were as follows: patients with (1) necrosis of the femoral head due to trauma or fracture, (2) hip dysplasia, femoral acetabular impingement and labral tears, (3) severe metabolic diseases, (4) tumour-related diseases and (5) a history of hip surgery. Finally, 101 patients (139 hips) were included and classified into two groups based on the surgical methods. Among these patients, 41 patients (59 hips) were treated with endoscopic intracapsular decompression combined with intramedullary decompression from July 2017 to December 2019 (group A), and 60 patients (80 hips) received small-diameter multi-channel core decompression alone from January 2015 to June 2017 (group B). For evaluation, all patients underwent pelvic anteroposterior and frog lateral X-ray and magnetic resonance imaging (MRI) of the hip joint before surgery. All patients included in the study were evaluated for the necrotic area and location according to Japanese Investigation Committee (JIC) classification criteria and the modified Kerboul angle classification system [7, 22]. The ARCO stage, JIC type and modified Kerboul angle classification system of ONFH was determined by two specialists based on the patients’ symptoms, X-ray and MR images. If the opinions of two experts are different, we will intervene in the third expert’s staging opinion, so as to complete the preoperative staging of patients.

### General data

Age, gender ratio, body mass index (BMI), the reason of osteonecrosis, ARCO stage, JIC types and modified Kerboul angle of osteonecrosis, unilateral or bilateral lesion and preoperative visual analogue scale (VAS) and Harris scores of the two groups were recorded.

Group A comprised 25 men (37 hips) and 16 women (22 hips) aged 18–65 (average, 35.5 ± 9.8) years, with an average BMI of 23.5 ± 2.3 kg/m^2^. Unilateral lesions were found in 23 patients and bilateral lesions in 18 patients. A total of 11 alcoholic ONFH, 16 hormonal ONFH and 14 idiopathic ONFH cases were found. For ARCO staging, 18 hips were in stage I and 41 in stage II. By the JIC classification, there were 29 cases of Type A, 22 cases of Type B, and 8 cases of Type C. According to the modified Kerboul angle classification, 21 cases were ≤ 190°, 31 cases were 190°–240°, and 7 cases were ≥ 240°. The preoperative VAS score was 6.3 ± 2.1, and the Harris score was 59.5 ± 11.6.

Group B comprised 34 men (48 hips) and 26 women (32 hips) aged 19–65 (average, 37.7 ± 10.5) years, with an average BMI of 24.7 ± 1.8 kg/m^2^. Unilateral lesions were found in 40 patients and bilateral lesions in 20 patients. A total of 17 alcoholic ONFH, 23 hormonal ONFH and 20 idiopathic ONFH cases were found. For ARCO staging, 21 hips were in stage I and 59 in stage II. By the JIC classification, there were 37 cases of Type A, 28 cases of Type B, and 15 cases of Type C. According to the modified Kerboul angle classification, there were 26 cases of ≤ 190°, 42 cases of 190°-240°, and 12 cases of ≥ 240°. The preoperative VAS score was 6.6 ± 2.5, and the Harris score was 57.7 ± 9.5.

### Surgical methods and treatment

**Fig. 1:**
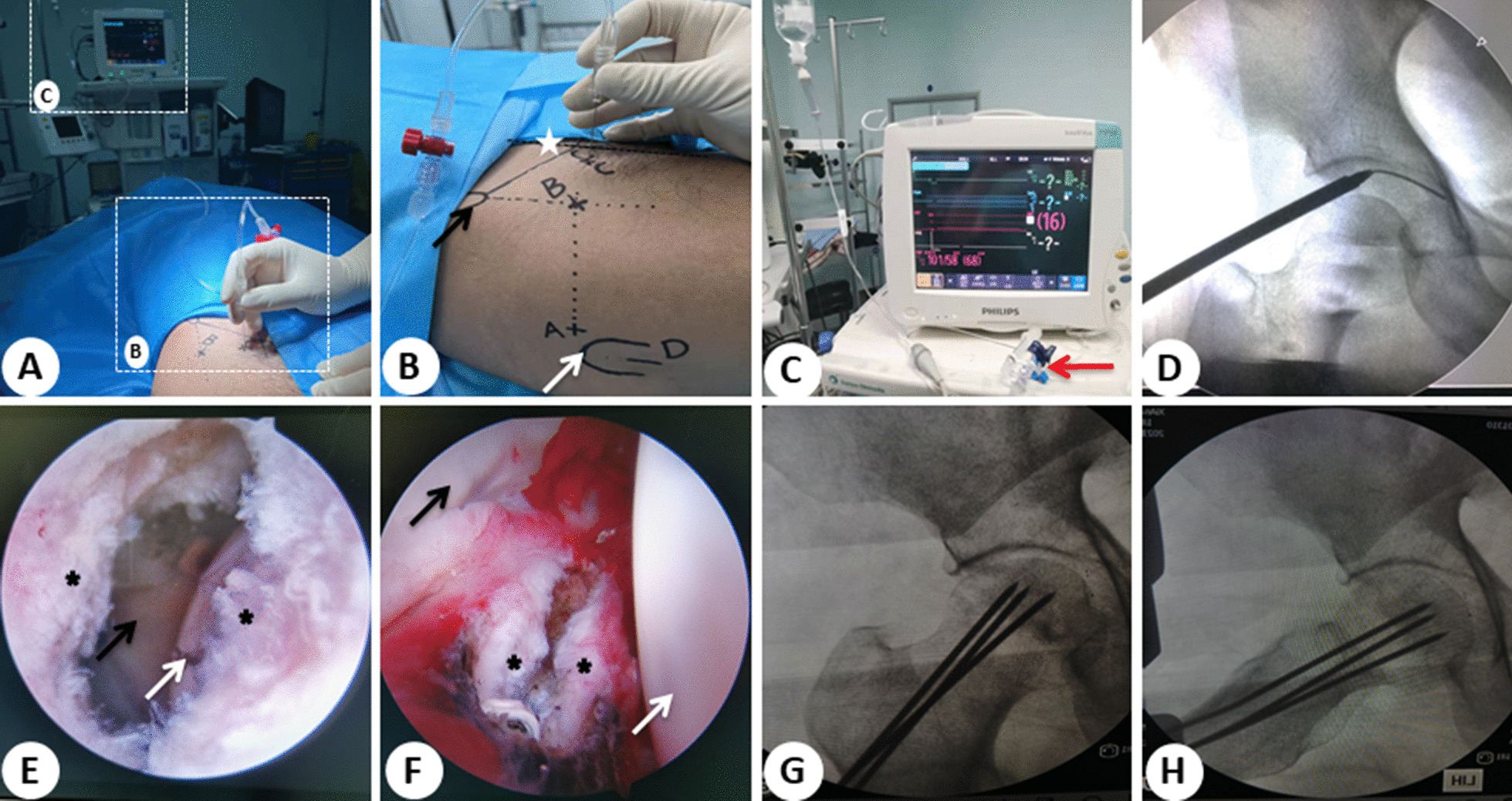
Measurement of intra-articular pressure and surgical operation. **A** Measurement of intra-articular pressure. **B** Bony landmarks and approaches of the hip joint (A/B/C/D symbol indicate the anterolateral approach, the anterior approach, the puncture point of intra-articular pressure measurement and the approach of core decompression, respectively. The black arrow indicates the anterior superior spine, the white arrow indicates the greater trochanter, and the white pentacle indicates the route of femoral vascular and nerve). **C** AI-4423 pressure sensor and IntelliVue MP50 pressure recorder. (The red arrow is a pressure sensor connected to the pressure detector.) **D** Establishment of the anterolateral approach for hip arthroscopy. **E** Incised lateral joint capsule outside the joint capsule through the view of the anterolateral approach. (The black pentacle shows the open joint capsule viewed from the outside, the black arrow represents the labrum, and the white arrow represents the femoral head.) **F** Incised lateral joint capsule inside the joint capsule through the view of the anterior approach. (The black pentacle shows the open joint capsule viewed from the inside, the black arrow represents the labrum, and the white arrow represents the femoral head.) **G**, **H** Intraoperative X-ray fluoroscopy of core decompression in the positive and frog positions

In both groups, surgery was performed under general anaesthesia. The bony landmarks of the hip joint, course of the blood vessels and nerves and entrance of arthroscopic instruments were marked before surgery (Fig. 1B). IAP was determined before disinfection and laying of sterile sheets using the method previously described in the literature [23]. The needle was inserted into the hip joint capsule from the lateral femoral artery of the distal groin for a hip puncture (Symbol C in Fig. 1B). The puncture needle was connected with an AI-4423 pressure sensor (Biomatrix, Israel) and IntelliVue MP50 pressure recorder (Philips, German). Normal saline was used as medium to generate pressure, and IAP was measured at a neutral position (Fig. 1A–C).

In group A, the patients laid flat with mild abduction and internal rotation of the affected limb and flexion as well as abduction and external rotation of the contralateral lower limb. After routine disinfection and laying of sterile sheets, a small skin incision (1 cm) was made at a site anterior to the top of the greater trochanter of the affected hip (Symbol A in Fig. 1B), and the hip joint was punctured using a long needle. Under C-arm X-ray guidance, a hip puncture was performed from an anterolateral portal (Fig. 1D). After entering the joint, arthroscopic access was established by expanding the casing step by step, and the anterior portal of capsule was made by sharp knife under arthroscopic monitoring (Symbol B in Fig. 1B). The synovial tissue with hyperplasia, hypertrophy, hyperaemia and oedema were cleaned by arthroscopic planning or radiofrequency vaporisation by the two portals alternating with arthroscopic monitoring. Then, the joint capsule of the anterolateral portal was expanded and cut open for approximately 1.5 cm without suturing (Fig. 1E and F), and a negative-pressure drainage tube was placed through this opening. Finally, the hip joint was rinsed with normal saline, the residual fluid in the joint cavity was drawn out and the arthroscope was removed. Subsequently, core decompression was performed and the surgical steps were similar to those in group B.

In group B, under C-arm X-ray guidance combined with preoperative X-ray and MRI or arthroscopy findings, the location of the necrotic area was identified and the drilling direction, position and depth were determined. From a site 2 cm below the greater trochanter of the femur (Symbol D in Fig. 1B), a 3.0-mm Kirschner wire was drilled into the osteonecrotic area in a fan shape in multiple directions (generally 2–5 directions) (Fig. 1G and 1H). When the drilling was close to the necrotic area, the electric drill was stopped and the Kirschner wire was hammered to reach a site 3.0–5.0 mm under the cartilage. This was done to avoid necrosis of bone cells in the peripheral wall of the femoral head tunnel due to the high amount of heat produced by the high-speed rotation and friction of the drill bit. A hard bone could be felt in the necrotic area. The procedure should be performed carefully to prevent the Kirschner wire from penetrating the cartilage surface of the femoral head.

After surgery, the IAP of the hip joint at the neutral position was measured using the same method in both groups.

### Post-operative treatment and follow-up

In group A, the negative-pressure drainage tube was withdrawn within 48 h after surgery. In both groups, the affected limbs could not bear weight within 6 weeks after surgery and used two crutches or wheelchairs for activities within 12 weeks and continued to maintain no weight bearing; during this period (6–12 weeks), patients can prevent joint stiffness by performing non-weight-bearing activities while in bed, began bearing partial weight (with crutches) 3 months after the surgery and then bore full weight gradually. At 1 and 4 weeks after surgery, the patients were followed up to evaluate effects and complications by senior surgeons (ZBS and LHF) in clinics or via telephone or email every month for 2 years. X-ray examination was performed every month in outpatient service or other hospitals (some patients received examination in other hospitals, and X-rays were collected by email) to observe the collapse of the femoral head within 2 years after surgery.

### Index evaluation

The surgery-related indexes of the two groups were compared, including IAP before and after surgery; length of hospital stay; hospitalisation expenses; VAS scores before surgery, 1 week, 4 weeks, 3 months and 1 year after surgery; and Harris scores of the hip joint before surgery, 3 months and 1 year after surgery. The follow-up results of all patients were recorded. The proportion of patients in the two groups without femoral head collapse (ARCO stage III and above) within 2 years after surgery was compared using Kaplan–Meier survival curve analysis.

### Statistical methods

Statistical analysis was conducted using IBM SPSS 24.0 (IBM SPSS Inc., Chicago, USA). The normally distributed measurement data were expressed as mean ± standard deviation (x ± s) and compared using the *t* test. The enumeration data were expressed as percentages (%) and compared using the chi-square test. The non-collapse rate of the femoral head was compared between the two groups using the Kaplan–Meier survival curve. *P* < 0.05 was considered statistically significant.

## Results

### Comparison of general data

General data, including mean age, gender ratio, BMI, the type of osteonecrosis, ARCO stage, JIC classification and modified Kerboul angle of osteonecrosis, unilateral or bilateral lesion, preoperative VAS and Harris scores of the two groups were not significantly different between the two groups (*P* > 0.05) (Table 1).

**Table 1:** Comparison of general data between the two groups

Characteristics	Group A	Group B	*t*/*χ*^2^	*P*
Total number (patients/hips), *n*	41/59	60/80	0.0825	0.7739
Gender (male/female), *n*	25/16	34/26	0.1862	0.6661
Age (years), mean ± SD	35.5 ± 9.8	37.7 ± 10.5	1.0621	0.2908
BMI (mean ± SD, kg/m^2^)	23.5 ± 2.3	22.9 ± 2.6	1.1925	0.2359
Type (alcohol/steroid/idiopathic), *n*	11/16/14	17/23/20	0.1566	0.9863
ARCO Stage (I/II), *n*	18/41	21/59	0.3051	0.5807
Unilateral/bilateral, *n*	23/18	40/20	1.1593	0.2816
VAS, mean ± SD	6.3 ± 2.1	6.6 ± 2.5	0.7474	0.4561
Harris score, mean ± SD	59.5 ± 11.6	57.7 ± 9.5	1.0046	0.3168
JIC (Type A/B/C)	29/22/8	37/28/15	3.7704	0.1518
*Modified Kerboul angle*			0.3403	0.8435
≤ 190°	21	26		
190°–240°	31	42		
≥ 240°	7	12		

### Comparison of surgery-related indexes

The surgical duration in group A was 84.5 ± 15.8 min, which was significantly longer than that in group B (52.6 ± 12.5 min), showing a significant difference (*t* = 13.2850, *P* = 0.000). The hospitalisation expenses in group A increased significantly compared with group B [(15,050.4 ± 1805.2) CNY vs (9890.9 ± 2156.8) CNY, *t* = 14.9176, *P* = 0.000]. However, no significant differences were found in intraoperative bleeding or the length of post-operative hospital stay between the two groups (*P* > 0.05) (Table 2).

**Table 2:** Comparison of surgery-related indexes

Items	Group A (*n* = 59)	Group B (*n* = 80)	*T*	*P*
Surgical duration (min)	84.5 ± 15.8	52.6 ± 12.5	13.2850	**0.0000**
Intraoperative bleeding (mL)	128.4 ± 26.2	119.8 ± 31.7	1.6990	0.0916
Hospital stay time (d)	5.5 ± 1.7	5.8 ± 2.1	0.9008	0.3693
Hospitalisation expenses (CNY)	15,050.4 ± 1805.2	9890.9 ± 2156.8	14.9176	**0.0000**

Bold idicates all *P* values with statistical differences

When the preoperative and post-operative pressure values in the hip joint capsule were compared, no significant differences were found in the preoperative pressure between the two groups (*P* > 0.05). In group A, the pressure in the hip joint capsule after surgery was significantly lower than that before surgery [(7.3 ± 6.1) mmHg vs. (25.5 ± 10.5) mmHg, *t* = 11.9136, *P* = 0.000]. In group B, the preoperative and post-operative pressure values in the hip joint capsule were not significantly different (*P* > 0.05). There were also statistically differences in post-operative IPA between the two groups (Table 3).

**Table 3:** Comparison of preoperative and post-operative pressure values in the hip joint capsule in each group and between the two groups

	A (*n* = 59)	B (*n* = 80)	*T*	*P*
Preoperative IAP (mmHg)	25.5 ± 10.5	24.2 ± 11.5	0.6944	0.4886
Post-operative IAP (mmHg)	7.3 ± 6.1	26.8 ± 7.8	15.9372	**0.0000**
*T*	11.9136	1.4725		
*P*	**0.0000**	0.1432		

Bold idicates all *P* values with statistical differences

### Comparison of preoperative and post-operative VAS and Harris scores of the hip joint between the two groups

No significant differences were found in preoperative VAS or Harris scores between the two groups (*P* > 0.05). After surgery, the VAS and Harris scores in both the groups were significantly improved compared with those before surgery, which was more evident in group A. At 1 week, 4 weeks, 3 months and 1 year after surgery, the VAS in group A significantly reduced compared with those in group B, which showed significant differences (Table 4). At 3 and 12 months after surgery, the Harris scores in group A significantly increased compared with those in group B, showing significant differences (Table 5).

**Table 4:** Comparison of the VAS scores before, 1 week and 4 weeks after surgery between the two groups

Time	Group A (*n* = 59)	Group B (*n* = 80)	*T*	*P*
Preoperative	6.3 ± 2.1	6.6 ± 2.5	0.7474	0.4561
1 Week after surgery	1.3 ± 1.1	3.6 ± 1.7	9.0800	**0.0000**
4 Weeks after surgery	1.5 ± 1.6	3.8 ± 2.1	7.0377	**0.0000**
3 Months after surgery	2.9 ± 1.3	4.0 ± 2.2	3.4231	**0.0008**
12 Months after surgery	3.1 ± 1.7	3.9 ± 2.1	2.4021	**0.0176**

Bold idicates all *P* values with statistical differences

**Table 5:** Comparison of Harris scores before, 3 months and 12 months after surgery between the two groups

Time	Group A (*n* = 59)	Group B (*n* = 80)	*T*	*P*
Before surgery	59.5 ± 11.6	57.7 ± 9.5	1.0046	0.3168
3 Months after surgery	85.1 ± 7.7	77.2 ± 8.3	5.7176	**0.0000**
12 Months after surgery	81.9 ± 10.2	76.4 ± 9.1	3.3451	**0.0011**

Bold idicates all *P* values with statistical differences

### Comparison of post-operative complications and collapse

**Fig. 2:**
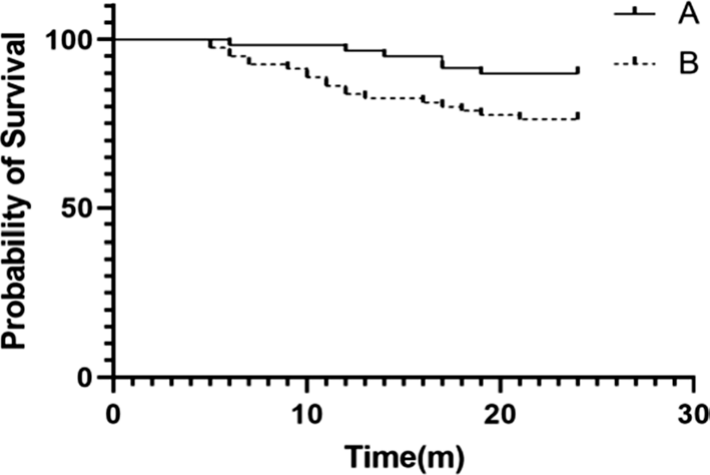
Comparison of Kaplan–Meier survival curves of the femoral head between the two groups. Line A shows the non-collapse rate of the femoral head in group A (treated with intramedullary core decompression combined with endoscopic intracapsular decompression). Line B shows the non-collapse rate of the femoral head in group B (treated with intramedullary core decompression alone). There were differences between the two groups (*P* < 0.05)

A follow-up of more than 2 years was conducted for both the groups. All incisions healed well. No vascular or nerve injury or other related complications occurred in all patients. X-ray examination revealed that 6 hips in group A and 22 hips in group B underwent femoral head collapse at the 2-year follow-up. All the 6 (6/41, 14.6%) collapsed patients in group A are ARCO stage II. In group B, 4 (4/21, 19.0%) collapsed patients are ARCO stage I and 18 (18/59, 30.5%) are ARCO stage II collapses. In general, the non-collapse rate of early femoral head necrosis treated in group A [89.8% (53/59)] was significantly higher than that in group B [72.5% (58/80)] (*P* = 0.0352) (Fig. 2).

## Discussion

At present, there are many methods for hip preservation treatment of ONFH, but their advantages and disadvantages and indications are different, and the post-operative efficacy is also controversial. Sadile et al. conducted a meta-analysis of 12 studies with a total of 776 patients and found that core decompression did not significantly delay the occurrence of hip osteoarthritis compared with other hip preservation treatments and the choice of surgical modality should be based on the situation of venous stasis and artery insufficiency [24]. However, Migliorini et al. found that several hip-preserving surgical modalities (osteotomy; non-vascularised bone grafting; multiple epiphyseal drilling; and free vascularised fibular graft) were available and effective for ONFH in patients with skeletal immature [25]. At the same time, Marco’s recent study showed that about one-third of patients undergoes a THA by 7 years after the osteotomy, and THA after osteotomy failed has higher technical requirements [26]. As the most commonly used surgery, core decompression is used as an early intervention for ONFH. It is based on the theory of increased intramedullary pressure in osteonecrosis [23, 27]. Core decompression can reduce intraosseous pressure, improve intraosseous microcirculation, alleviate ischaemia and promote osteogenesis and bone repair [8]. Additionally, core decompression combined with bone marrow-derived cell therapies can effectively improve the regenerative situation of femoral head necrosis and reduce post-operative pain and lower rate of progression to THA [28]. A long-term follow-up study revealed that core decompression is safe and effective for treating early lesions, and its efficacy is higher than conservative treatment [29]. However, Camp et al. believed that the failure rate of surgery after core decompression treatment can reach 60% [30]. The first reason is that the supporting effect of the subchondral bone in the femoral head after large-diameter–single-channel drilling is weakened, which can easily cause femoral fractures and femoral head collapse at the drilling site [10]. Additionally, the traditional decompression method cannot achieve complete decompression at the osteonecrosis site because of the single decompression site, inaccurate positioning and incomplete removal of the dead bones [31]. Migliorini F et al. conducted a systematic review of 88 studies found that higher preoperative VAS and lower Harris score were negative factors affecting the clinical effect after core decompression, and the incidence of THA in female patients was significantly lower than that in male patients. These above factors have good similarity in this study [32].

In this study, the drilling decompression method with a small-diameter drill, multiple channels, multiple directions and low-speed rotation was employed. Small-diameter multi-channel core decompression is a modified method based on the previous large-channel core decompression. In this study, we applied the JIC classification to assess necrotic location and area for precise core decompression. During surgery, a 3.0-mm Kirschner wire was used to replace the traditional 8.0–10.0 mm drill bit for multi-channel decompression to change the overall decompression area and achieve the same effect. Song et al. used small-diameter multiple-channel core decompression for treating early and medium ONFH, with a 5-year success rate of 79.5% (31/39) in patients with stage I and 77% (62/81) in those with stage II disease and no secondary surgery [33]. Mont et al. conducted a 2 years follow-up study after using this technique and obtained a surgical success rate of 71.1% (32/45) [34]. Moreover, compared with the traditional core decompression, the presented technique resulted in a lower rate of post-operative femoral head collapse. In our study, the 2-year success rate of the small-diameter multi-channel core decompression was 72.5% (58/80), and no complications, such as fractures, infection and irreversible neurovascular injury, were observed after surgery, which was in agreement with the results of previous reports [18, 19].

The blood in the femoral head and neck is mainly supplied by the four groups of retinaculum arteries that originate from the medial and lateral circumflex arteries. These medial and lateral circumflex arteries pass through the attachment of the joint capsule at the femoral neck and supply more than 70% of the blood to the femoral head after entering the capsule [35, 36]. The increase in IAP can damage the blood flow to the femoral head and may lead to osteonecrosis [37]. Vidyadhar evaluated the correlation between IAP and epiphyseal perfusion pressure using a pig model and believed that decompression and IAP reduction could reduce the risk of avascular necrosis in patients with intra-articular proximal femoral fractures who underwent hip capsulotomy [15]. A meta-analysis study showed whether to suture the joint capsule has no significant effect on the post-operative function after hip arthroscopy [35]. Therefore, in the present study, the joint capsule was not sutured after hip arthroscopy to reduce the pressure in the hip joint. Further, the drainage tube was placed through the joint capsule for post-operative drainage to further reduce the IAP. Our study measured the IAP before and after surgery, revealing that the IAP decreased significantly after arthroscopy, which was not evaluated in recent studies [18, 19]. In this study, the post-operative VAS was significantly lower, and the hip functions also showed significant improvement and the 2-year survival rate of the femoral head significantly increased to 89.8% (53/59) in group A. All these results may be related to the decrease in intramedullary pressure and IAP of the femoral head, which increases the blood supply to the femoral head and promotes the venous reflux of the femoral head.

Hip arthroscopy can directly detect the internal environment of the hip joint and the surface damage of the femoral head and accurately evaluate the stage of ONFH [36]. Anil believed that hip arthroscopy could help surgeons accurately determine whether patients have an apparent joint injury, whether there is a possibility of continuous progression and whether patients need core decompression. Additionally, intra-articular synovitis can be detected in patients with ONFH using arthroscopy [18]. In the present study, we found similar lesions, suggesting that ONFH is a total joint disease. The synovium was cleaned by arthroscopic planning and radiofrequency ablation, and the inflammatory substances were removed with a large volume of normal saline, which can improve the intra-articular environment and reduce IAP [13, 17]. An MR study by Nazal et al. demonstrated that arthroscopic adjuvant therapy is a promising surgical method, which can provide safe, accurate and minimally invasive decompression to obtain reliable results and an acceptable conversion rate of THA [19].

However, the anterior approach of hip arthroscopy can easily damage the femoral arteries and nerves. The anterolateral approach is considered safe as it could avoid interaction with the lateral femoral cutaneous nerve. Thus, it can prevent the injury of blood vessels and nerves before surgery by certainly marking the body surface projection [38, 39]. Additionally, the soft tissue injury in the perineal area and cyanosis of the foot caused by prolonged instrument traction and compression should be noted [40]. However, in this study, we only performed arthroscopic debridement and joint capsule incision decompression and did not need traction to obtain a wider surgical space and avoid the generation of traction related complications. Moreover, our study showed that arthroscopy increased the surgical duration and cost.

This study also has several limitations. First, treatment with traditional core decompression was not set as the control group and the results of the present treatment were not compared with those of other treatments, such as bone transplantation with or without a vascular pedicle. Second, the total sample size was small, and the follow-up duration was short; thus, the reliability of the conclusion needs to be further confirmed by studies with larger sample sizes and longer follow-up duration. Third, in arthroscopic surgery, the joint space should be pulled by 8–10 mm and the puncture needle, guidewire, arthroscope and surgical instruments should be introduced into the joint cavity; thus, it is peculiarly prone to arthroscopy-related complications. Fourth, the size of the joint capsule incision may affect IAP after surgery. The smaller the incision, the easier the healing would be. In addition, we only detected IAP immediately after surgery and did not continuously monitor IAP during later follow-ups. Fifth, further research is thought to be needed to prove the direct correlation between the decrease in IAP and better post-operative results. Finally, in this study, the preoperative staging of osteonecrosis of the femoral head was judged only by imaging data, and ONFH was not re-staging under arthroscopy, which needs to be improved in further prospective studies.

## Conclusion

In conclusion, small-diameter multi-channel core decompression combined with intra-articular decompression (debridement of the hip joint and incision of the hip capsule) under hip arthroscopic guidance is a simple and minimally invasive surgery in the treatment of early ONFH, which can more effectively alleviate pain, improve joint function and delay the development of ONFH. The mid-term follow-up results are good, and the long-term follow-up results need further observation.

## Data Availability

The datasets used during the current study are available from the corresponding author on reasonable request.

## Abbreviations

ONFH: Non-traumatic osteonecrosis of the femoral head
THA: Total hip arthroplasty
IAP: Intra-articular pressure
VAS: Visual analogue scale

## References

[1] Konarski W, Poboży T, Śliwczyński A, Kotela I, Krakowiak J, Hordowicz M, Kotela A (2022). Avascular necrosis of femoral head-overview and current state of the art. Int J Environ Res Public Health.

[2] Betsch M, Tingart M, Driessen A, Quack V, Rath B (2018). Total hip replacement in avascular femoral head necrosis. Orthopade.

[3] Cao H, Guan H, Lai Y, Qin L, Wang X (2016). Review of various treatment options and potential therapies for osteonecrosis of the femoral head. J Orthop Translat.

[4] Guggenbuhl P, Robin F, Cadiou S, Albert JD (2021). Etiology of avascular osteonecrosis of the femoral head. Morphologie.

[5] Moya-Angeler J, Gianakos AL, Villa JC, Ni A, Lane JM (2015). Current concepts on osteonecrosis of the femoral head. World J Orthop.

[6] Hines JT, Jo WL, Cui Q, Mont MA, Koo KH, Cheng EY, Goodman SB, Ha YC, Hernigou P, Jones LC (2021). Osteonecrosis of the femoral head: an updated review of ARCO on pathogenesis, staging and treatment. J Korean Med Sci.

[7] Ando W, Sakai T, Fukushima W, Kaneuji A, Ueshima K, Yamasaki T, Yamamoto T, Nishii T, Working group for ONFH guidelines (2021). Japanese Orthopaedic Association 2019 Guidelines for osteonecrosis of the femoral head. J Orthop Sci.

[8] Pierce TP, Jauregui JJ, Elmallah RK, Lavernia CJ, Mont MA, Nace J (2015). A current review of core decompression in the treatment of osteonecrosis of the femoral head. Curr Rev Musculoskelet Med.

[9] Mont MA, Ragland PS, Etienne G (2004). Core decompression of the femoral head for osteonecrosis using percutaneous multiple small-diameter drilling. Clin Orthop Relat Res.

[10] Brown PJ, Mannava S, Seyler TM, Plate JF, Van Sikes C, Stitzel JD, Lang JE (2016). Multiple small diameter drillings increase femoral neck stability compared with single large diameter femoral head core decompression technique for avascular necrosis of the femoral head. Surg Technol Int.

[11] Beck M, Siebenrock KA, Affolter B, Notzli H, Parvizi J, Ganz R (2004). Increased intraarticular pressure reduces blood flow to the femoral head. Clin Orthop Relat Res.

[12] Sotohall R, Johnson LH, Johnson RA (1964). Variations in the intra-articular pressure of the hip joint in injury and diease—a probable factor in avascular necrosis. J Bone Jt Surg - Am.

[13] Zlotorowicz M, Szczodry M, Czubak J, Ciszek B (2011). Anatomy of the medial femoral circumflex artery with respect to the vascularity of the femoral head. J Bone Jt Surg - Br.

[14] Crock HV (1980). An atlas of the arterial supply of the head and neck of the femur in man. Clin Orthop Relat Res.

[15] Upasani VV, Badrinath R, Farnsworth CL, Jeffords ME, Hallare JA, Ahmed SI, Schrader T (2020). Increased hip intracapsular pressure decreases perfusion of the capital femoral epiphysis in a skeletally immature porcine model. J Pediatric Orthop.

[16] Yen CH, Leung HB, Tse PY (2009). Effects of hip joint position and intra-capsular volume on hip joint intra-capsular pressure: a human cadaveric model. J Orthop Surg Res.

[17] Wirth T (2006). Coxitis fugax—the beginning of Perthes' disease?. Z Orthop Ihre Grenzgeb.

[18] Gupta AK, Frank RM, Harris JD, McCormick F, Mather RC, Nho SJ (2014). Arthroscopic-assisted core decompression for osteonecrosis of the femoral head. Arthrosc Tech.

[19] Nazal MR, Parsa A, Martin SD (2019). Mid-term outcomes of arthroscopic-assisted core decompression of precollapse osteonecrosis of femoral head-minimum of 5 year follow-up. BMC Musculoskelet Disord.

[20] Murphey MD, Roberts CC, Bencardino JT, Appel M, Arnold E, Chang EY, Dempsey ME, Fox MG, Fries IB, Greenspan BS (2016). ACR appropriateness criteria osteonecrosis of the hip. J Am Coll Radiol.

[21] Choi H-R, Steinberg ME, Cheng EY (2015). Osteonecrosis of the femoral head: diagnosis and classification systems. Curr Rev Musculoskelet Med.

[22] Ha Y, Jung W, Kim J, Seong N, Kim S, Koo K (2006). Prediction of collapse in femoral head osteonecrosis: a modified Kerboul method with use of magnetic resonance images. J Bone Jt Surg Am.

[23] Hungerford DS, Lennox DW (1985). The importance of increased intraosseous pressure in the development of osteonecrosis of the femoral-head-implications for treatment. Orthop Clin N Am.

[24] Sadile F, Bernasconi A, Russo S, Maffulli N (2016). Core decompression versus other joint preserving treatments for osteonecrosis of the femoral head: a meta-analysis. Br Med Bull.

[25] Migliorini F, La Padula G, Oliva F, Torsiello E, Hildebrand F, Maffulli N (2022). Operative management of avascular necrosis of the femoral head in skeletally immature patients: a systematic review. Life (Basel).

[26] Quaranta M, Miranda L, Oliva F, Aletto C, Maffulli N (2021). Osteotomies for avascular necrosis of the femoral head. Br Med Bull.

[27] Rubio-Martinez LM, Carstens A (2013). Medullary decompression of the radius as treatment for lameness in a horse. Vet Comp Orthop Traumatol.

[28] Migliorini F, Maffulli N, Eschweiler J, Tingart M, Baroncini A (2021). Core decompression isolated or combined with bone marrow-derived cell therapies for femoral head osteonecrosis. Expert Opin Biol Ther.

[29] Etemadifar M, Kooskzari M, Khalilollah N, Ali MK, Mahsa B (2014). The results of core decompression treatment in patients with avascular necrosis of femoral head in patients at Isfahan City educational hospitals in 2010–2011. Adv Biomed Res.

[30] Camp JF, Colwell CW (1986). Core decompression of the femoral-head for osteonecrosis. J Bone Jt Surg - Am.

[31] Pierannunzii L (2012). Endoscopic and arthroscopic assistance in femoral head core decompression. Arthrosc Tech.

[32] Migliorini F, Maffulli N, Baroncini A, Eschweiler J, Tingart M, Betsch M (2022). Prognostic factors in the management of osteonecrosis of the femoral head: a systematic review. Surgeon.

[33] Song WS, Yoo JJ, Kim YM, Kim HJ (2007). Results of multiple drilling compared with those of conventional methods of core decompression. Clin Orthop Relat Res.

[34] Mont MA, Jones LC, Hungerford DS (2006). Nontraumatic osteonecrosis of the femoral head: ten years later. J Bone Jt Surg - Am.

[35] Kunze KN, Vadhera A, Devinney A, Nwachukwu BU, Kelly BT, Nho SJ, Chahla J (2021). Effect of capsular closure after hip arthroscopy for femoroacetabular impingement syndrome on achieving clinically meaningful outcomes: a meta-analysis of prospective and comparative studies. Orthop J Sports Med.

[36] Pierce TP, Jauregui JJ, Cherian JJ, Elmallah RK, Mont MA (2015). Imaging evaluation of patients with osteonecrosis of the femoral head. Curr Rev Musculoskelet Med.

[37] Li J, Li Z-L, Zhang H, Su X-Z, Wang K-T, Yang Y-M (2017). Long-term outcome of multiple small-diameter drilling decompression combined with hip arthroscopy versus drilling alone for early avascular necrosis of the femoral head. Chin Med J.

[38] Moeckel G, Labs K (2014). Complications in hip arthroscopy and follow-up therapy. Analysis over a 5-year time period with a total of 13,000 cases. Der Orthopade.

[39] Kowalczuk M, Bhandari M, Farrokhyar F, Wong I, Chahal M, Neely S, Gandhi R, Ayeni OR (2013). Complications following hip arthroscopy: a systematic review and meta-analysis. Knee Surg Sports Traumatol Arthrosc.

[40] Simpson J, Sadri H, Villar R (2010). Hip arthroscopy technique and complications. Orthop Traumatol - Surg Res.

